# Photonics-based real-time ultra-high-range-resolution radar with broadband signal generation and processing

**DOI:** 10.1038/s41598-017-14306-y

**Published:** 2017-10-23

**Authors:** Fangzheng Zhang, Qingshui Guo, Shilong Pan

**Affiliations:** Key Laboratory of Radar Imaging and Microwave Photonics, Ministry of Education, Nanjing University of Aeronautics and Astronautics, Nanjing, 210016 China

## Abstract

Real-time and high-resolution target detection is highly desirable in modern radar applications. Electronic techniques have encountered grave difficulties in the development of such radars, which strictly rely on a large instantaneous bandwidth. In this article, a photonics-based real-time high-range-resolution radar is proposed with optical generation and processing of broadband linear frequency modulation (LFM) signals. A broadband LFM signal is generated in the transmitter by photonic frequency quadrupling, and the received echo is de-chirped to a low frequency signal by photonic frequency mixing. The system can operate at a high frequency and a large bandwidth while enabling real-time processing by low-speed analog-to-digital conversion and digital signal processing. A conceptual radar is established. Real-time processing of an 8-GHz LFM signal is achieved with a sampling rate of 500 MSa/s. Accurate distance measurement is implemented with a maximum error of 4 mm within a range of ~3.5 meters. Detection of two targets is demonstrated with a range-resolution as high as 1.875 cm. We believe the proposed radar architecture is a reliable solution to overcome the limitations of current radar on operation bandwidth and processing speed, and it is hopefully to be used in future radars for real-time and high-resolution target detection and imaging.

## Introduction

Real-time and high-resolution target detection and imaging is of great importance in civil and security applications such as capturing and tracking fast moving targets, which requires a radio-frequency (RF) radar to be operated at a high frequency and a wide bandwidth with real-time signal processing capability^1,2^. This requirement creates great challenges to the state-of-the-art electronics. On one hand, in radar transmitters direct generation of linear frequency modulation (LFM) signals by means of direct digital synthesizers (DDS) is limited to a few gigahertz^3^. Although this bandwidth can be expanded by multiple stages of frequency up-conversion, the signal quality would be inevitably deteriorated which eventually affects the detection performance. On the other hand, the precision of analog-to-digital converters (ADCs) in the receiver drops rapidly as the input bandwidth and sampling rate increase, which severely restricts the resolution as well as the processing speed. Recently, microwave photonic technologies have been proposed as a promising solution for the generation, detection, and processing of high-frequency RF signals^3–6^, taking advantage of the high-frequency and broadband operation capability provided by optical components. Up to now, a lot of schemes for photonic generation of broadband LFM signals have been demonstrated^7–10^, where a signal bandwidth over 10 GHz can be easily achieved. However, fast and convenient processing of such broadband signals without sacrificing signal fidelity is still a difficult task. In a previously reported photonics-based fully digital coherent radar^11^, the great potential of photonic technologies in future radar applications is demonstrated, but the signal processing in the sampling receiver is still a main limitation of the operation frequency and bandwidth. To down-convert the high-frequency RF signals, microwave photonic frequency conversion and time-stretched analog-to-digital conversion techniques have been proposed^12–15^, but it is still hard for a traditional radar receiver to process the down-converted baseband or intermediate frequency (IF)-band signals if a very large operation bandwidth is adopted.

In this article, we propose and demonstrate a photonics-based real-time high-range-resolution radar incorporating optical generation and processing of broadband LFM signals. In the transmitter, a broadband LFM signal is generated by frequency quadrupling of a low-speed electrical signal applying a single integrated electro-optical modulator. In the receiver, the reflected LFM signal is de-chirped to a low-frequency signal based on photonic frequency mixing. The implementation of photonic de-chirping can directly process high-frequency and large bandwidth signals without any electrical frequency conversion. After photonic de-chirping, ADC with a moderate sampling rate can be used in the receiver and real-time signal processing is realizable. In the proposed system, the bandwidth limitations due to electrical signal generation and processing is eliminated. The maximum operation bandwidth is mainly determined by the electro-optical devices, which can be tens or even hundreds of gigahertz. As a result, real-time radar detection with a very high range resolution can be realized.

## Results

### Principle and system design

**Figure 1:**
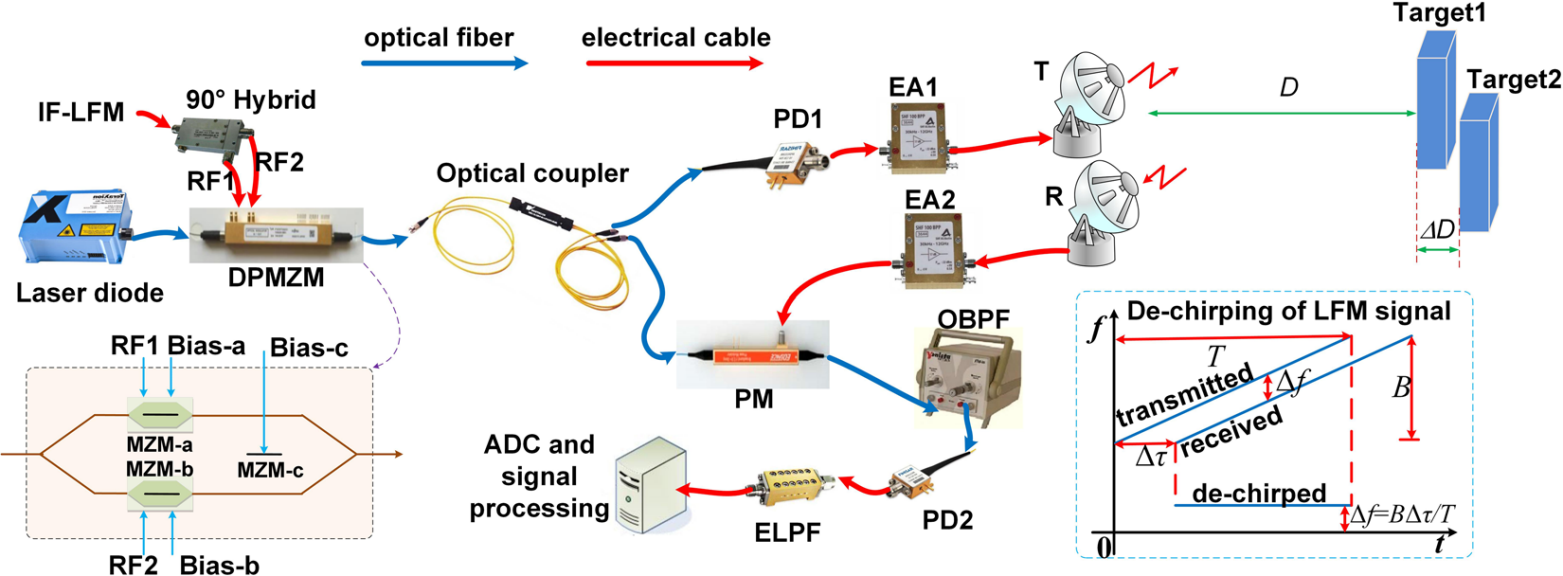
Setup of the proposed photonics-based radar. DPMZM: dual-parallel Mach-Zehnder modulator; PD: photodetector; PM: electro-optical phase modulator; OBPF: optical bandpass filter; ELPF: electrical low-pass filter; ADC: analog-to-digital conversion. Inset: principle for de-chirping of a LFM signal.

Figure 1 shows the schematic diagram of the proposed photonics-based radar system. A continuous wave light from a laser diode is modulated by a dual-parallel Mach-Zehnder modulator (DPMZM) that is driven by a continuous-wave IF-band LFM signal generated by a low-speed electrical signal generator. For ease of understanding, we assume the instantaneous frequency of the IF-LFM signal is *f*_IF_(*t*) = *f*_0_ + *kt*, where *f*_0_ is the initial frequency and *k* is the linear chirp rate. The DPMZM consists of two sub-MZMs (MZM-a and MZM-b), and each sub-MZM is embedded in one arm of the parent MZM (MZM-c). Before applied to the DPMZM, the IF-LFM signal passes through an electrical 90° hybrid, and the obtained two signals with 90° phase difference are used to drive the two sub-MZMs, respectively. By properly setting the bias voltages of the DPMZM, only the ±2^nd^ order modulation sidebands at frequencies of *f*_c_ − 2*f*_0_ − 2*kt* and *f*_c_ + 2*f*_0_ + 2 *kt* are generated, where *f*_c_ is the frequency of the laser source. This optical signal is then equally split into two branches by an optical coupler (OC). In one branch, the optical signal is used as a reference for de-chirp processing of the received echoes, and in the other branch, the signal is sent to a photodetector (PD1) to implement optical-to-electrical conversion. The obtained electrical signal has a frequency that is quadruped compared to that of the IF-LFM signal. Consequently, a frequency-quadrupled LFM signal is obtained with an instantaneous frequency of *f*_LFM_(*t*) = 4*f*_0_ + 4 *kt*. The LFM signal is amplified by a broadband electrical amplifier (EA1) and launched into the air through an antenna for target detection. The signal reflected by the target is collected by another antenna and properly amplified by EA2 before applied to an electro-optical phase modulator (PM). The PM is used to modulate the reference optical signal from the lower branch of the OC. In this process, the two optical sidebands in the reference optical signal can be treated as two optical carriers at *f*_c_ − 2*f*_0_ − 2*kt* and *f*_c_ + 2*f*_0_ + 2 *kt*, and they are phase modulated by the reflected LFM signal. The frequency of the 1^st^-order sideband generated by phase modulating the carrier at *f*_c_ − 2*f*_0_ − 2*kt* is located at *f*_c_ + 2*f*_0_ + 2 *kt* + 4*k*Δ*τ*, where Δ*τ* is the time delay of the reflected LFM signal compared with the transmitted signal. By properly designing the parameters of the transmitted LFM signal according to the detection range to let 4*k*Δ*τ* be a small value, this 1^st^-order sideband is very close to the optical carrier at *f*_c_ + 2*f*_0_ + 2 *kt*, and they can be extracted using an optical bandpass filter (OBPF). Before the OBPF, an erbium-doped optical fiber amplifier (EDFA) can be applied to boost the optical power. After the OBPF, the optical signal is sent to another photodetector (PD2) to perform optical-to-electrical conversion. An electrical signal with a frequency of Δ*f* = 4*k*Δ*τ* is obtained. To avoid the high-frequency interference, an electrical low-pass filter (ELPF) with a proper bandwidth can be applied after PD2. To this point, photonic de-chirping of the received LFM signal based on photonic frequency mixing is completed. In practice, this de-chirped frequency can be acquired by sampling the de-chirped signal using a low-speed electrical ADC and then performing simple spectral analysis. Considering 4*k* is the chirp rate of the transmitted LFM signal, the time delay Δ*τ* can be expressed as

1 $${\rm{\Delta }}\tau =\frac{{\rm{\Delta }}f}{4k}=\frac{T{\rm{\Delta }}f}{B}$$

where *B* is the bandwidth and *T* is the temporal period of the LFM signal transmitted through the antenna. The distance of the target is

2 $$L=\frac{{\rm{\Delta }}\tau }{2}c=\frac{c}{2B}T{\rm{\Delta }}f$$

The minimum spectral spacing that can be distinguished is Δ*f*_min_ = 1/*T*, thus the range-resolution is

3 $${L}_{{\rm{RES}}}=\frac{c}{2B}T{\rm{\Delta }}{f}_{{\rm{\min }}}=\frac{c}{2B}$$

Equation (3) indicates that a large bandwidth of the transmitted signal helps to achieve a high-range resolution. In the proposed radar, photonic generation and de-chirping of LFM signals can have a very large operation bandwidth. After photonic de-chirping, ADC with a moderate sampling speed and a high precision can be used in the receiver, which makes it possible for real-time signal processing. Therefore, high resolution real-time target detection can be achieved. In this paper, we focus on the characterization of the proposed photonics-based radar in the static targets detection. For scenarios of a moving antenna or a moving target, synthetic aperture radar (SAR) imaging or inverse synthetic aperture radar (ISAR) imaging can be performed^16,17^, which makes the proposed radar system very promising in various applications.

### Broadband LFM Signal generation

**Figure 2:**
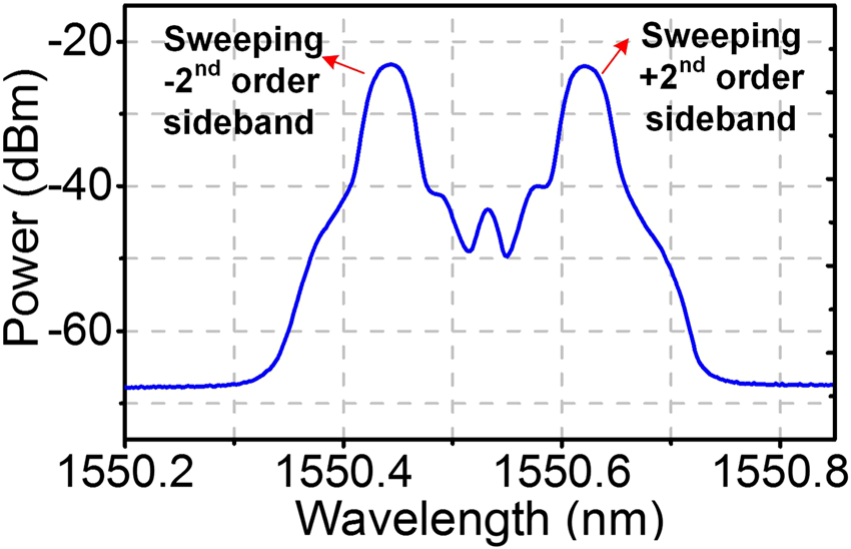
Measured optical spectrum after the DPMZM. Two frequency sweeping sidebands are generated.

**Figure 3:**
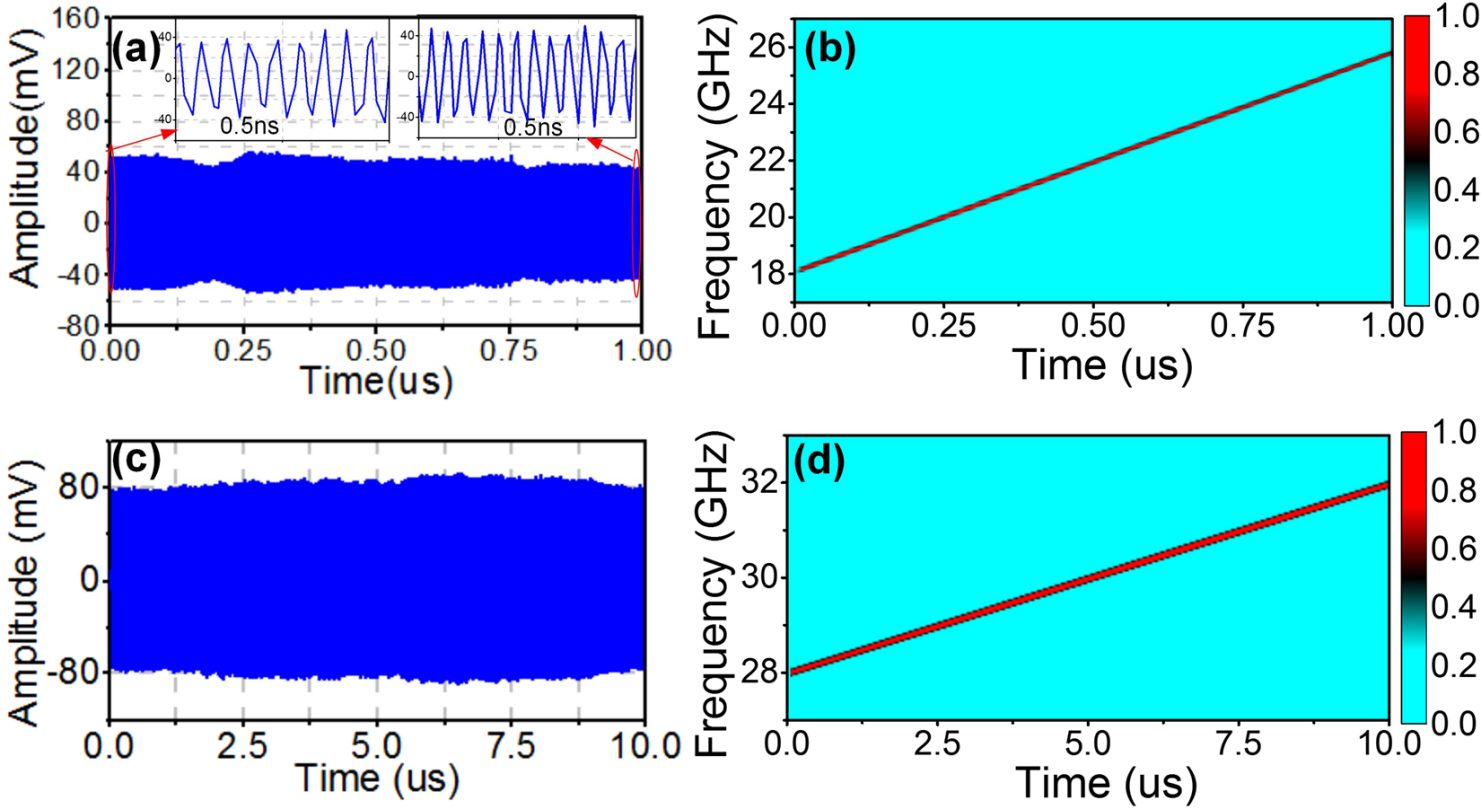
Results of broadband and reconfigurable LFM signal generation. (**a**) and (**b**): waveform and frequency of the generated 8-GHz LFM signal with a central frequency of 22 GHz and a repetition rate of 1 MHz; (**c**) and (**d**): waveform and frequency of the generated 4-GHz LFM signal with a central frequency of 30 GHz and a repetition rate of 100 kHz.

As an example, a 1-MHz repetition rate continuous wave IF-LFM signal centered at 5.5 GHz with a bandwidth of 2 GHz is applied to drive the DPMZM. After carefully setting the bias voltages of the DPMZM, frequency quadrupling of the input IF-LFM signal is realized. Figure 2 shows the optical spectrum after the DPMZM, where two frequency sweeping optical sidebands (the ±2^nd^ order sidebands) are generated with the undesired sidebands well suppressed. Figure 3(a) shows the measured waveform of the generated LFM signal in one period (1 μs). Frequency variation of the LFM signal can be easily observed by comparing the waveforms in the insets of Fig. 3(a). Figure 3(b) shows the instantaneous frequency corresponding to the waveform in Fig. 3(a), which is achieved by applying short-time Fourier transform (STFT) analysis. As can be seen, the frequency is in the range from 18 GHz to 26 GHz and the signal bandwidth is 8 GHz, confirming the frequency quadrupling capability. Bandwidth and repetition rate of the generated LFM signal can be easily adjusted by changing the parameters of the IF-LFM signal, which is feasible for the current low-speed electrical signal generators. Thus, reconfigurable LFM signal generation is feasible. Figure 3(c) shows the temporal waveform of the generated LFM signal when the IF-LFM signal is centered at 7.5 GHz with a bandwidth of 1 GHz and a repetition rate of 100 kHz. The corresponding frequency shown in Fig. 3(d) covers from 28 GHz to 32 GHz with a temporal period of 10 μs.

### De-chirping of radar echoes

**Figure 4:**
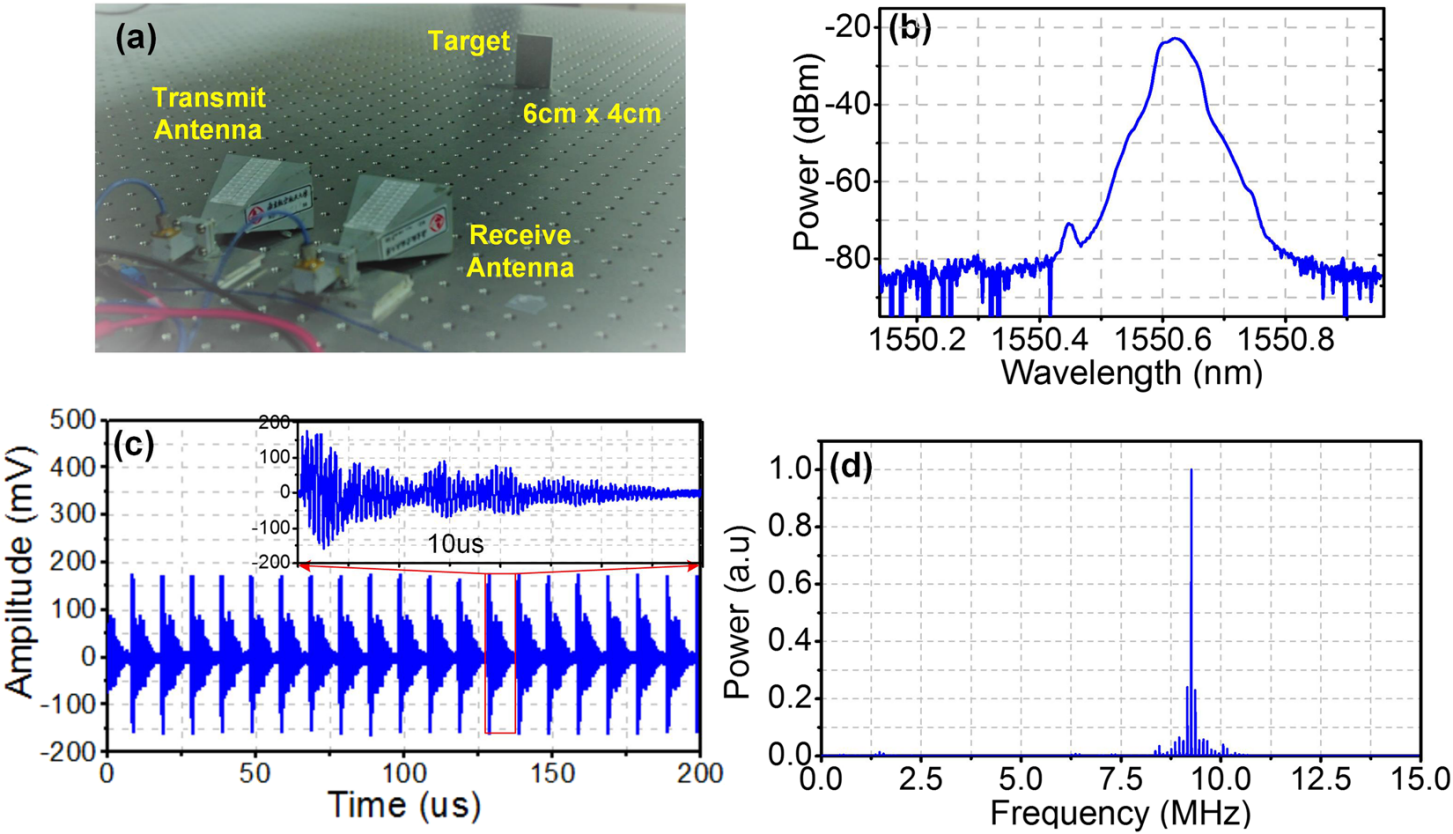
(**a**) Configuration for single target detection; (**b**) optical spectrum after OBPF; (**c**) temporal waveform of de-chirped signal when the distance between the target and the antenna pair is 173.6 cm; (**d**) normalized power spectrum of the de-chirped signal with a spectral peak at 9.253 MHz.

De-chirping of a K-band LFM signal with a repetition rate of 100 kHz and bandwidth of 8 GHz (from 18 GHz to 26 GHz) is demonstrated. The LFM signal is amplified and sent to a K-band horn antenna for air transmission toward a plane metallic target with a size of about 6 cm × 4 cm, as shown in Fig. 4(a). In this demonstration, the target is placed at a distance of 173.6 cm away from the transmit antenna. The reflected signal is collected by another K-band horn antenna placed close to the transmit antenna. The collected signal is amplified by another broadband amplifier before sent to the RF port of a 40-GHz PM. After the PM, the optical signal is properly amplified by an EDFA. Then, a bandwidth and frequency tunable OBPF is applied to select the required optical frequency components. The optical spectrum after the OBPF is shown in Fig. 4(b). This optical signal is sent to a 10-GHz PD (PD2) and the generated electrical signal passes through an ELPF with a 3-dB bandwidth of 500 MHz. Then, the de-chirped signal is digitalized by a real-time oscilloscope and processed by the same oscilloscope which can perform real-time spectral analysis based on fast Fourier transform (FFT). The de-chirped signal in a period of 200 μs is captured with a sampling rate of 500 MSa/s and then processed in real time. Figure 4(c) shows the recorded waveform with a detailed waveform shown in the inset. The normalized power spectrum of the de-chirped signal is shown in Fig. 4(d), where the spectral peak at 9.253 MHz corresponds to the dominate frequency of the de-chirped signal. In obtaining the power spectrum in Fig. 4(d), a simple calibration has been done to remove the frequency offset due to time delay of the electrical cables and other devices, so that the de-chirped frequency is proportional to the time delay between the target and the antenna pair.

### Radar target detection

**Figure 5:**
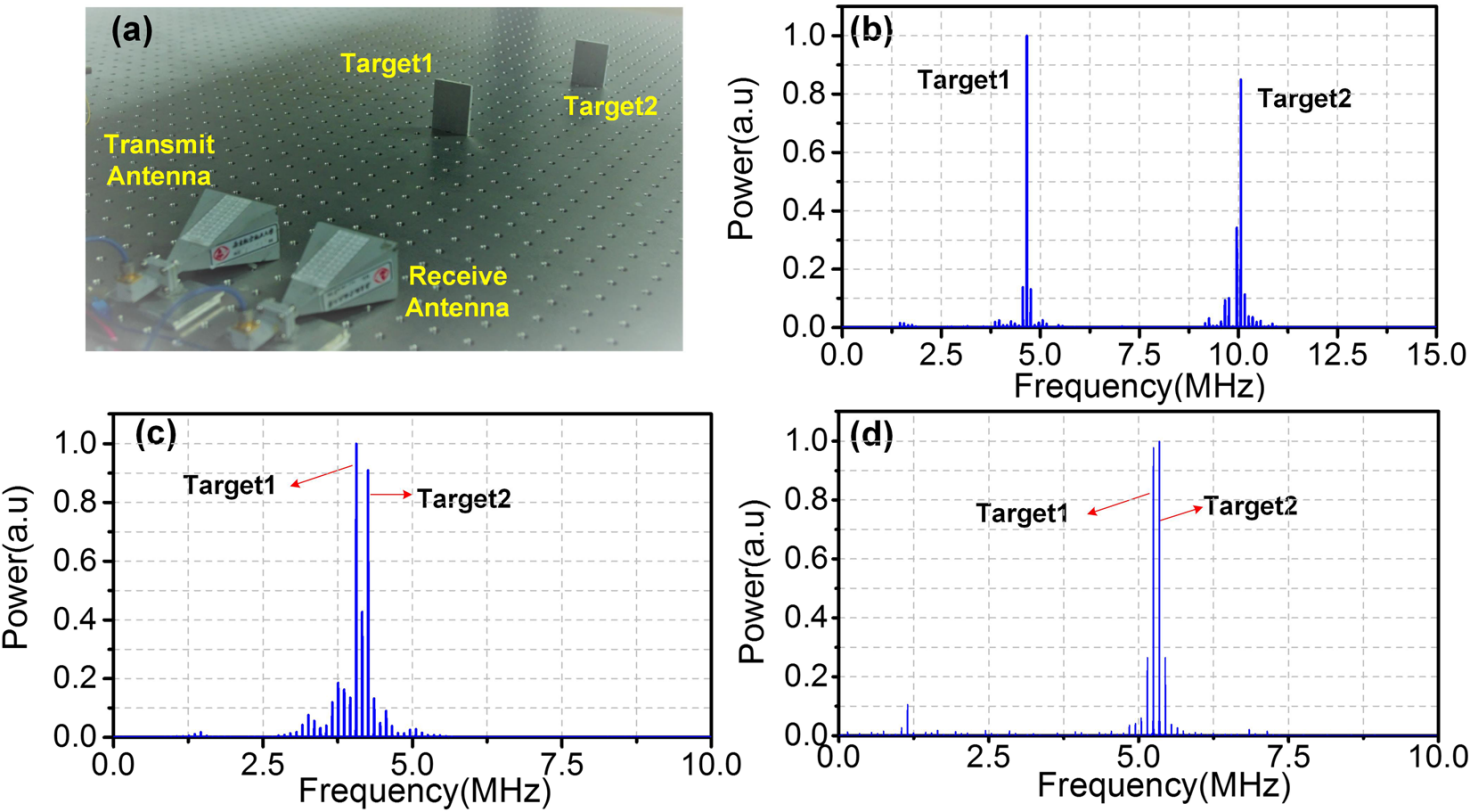
(**a**) System diagram for detection of two targets; (**b**) power spectrum of de-chirped signal when positions of the two targets are 87.4 cm and 188 cm; (**c**) power spectrum of de-chirped signal when the two targets are separated by 3.8 cm; (**d**) power spectrum of de-chirped signal when the two targets are separated by 1.9 cm.

In the case corresponding to Fig. 4(c) and (d), the distance between the target and the antenna pair is calculated to be 173.5 cm and the measurement error is 1 mm. By changing the target position, multiple distance measurements are implemented. The maximum distance is set as large as the experimental condition permits. The measurement results are: (52.0 cm, 51.7 cm), (79.5 cm, 79.8 cm), (128.3 cm, 128.5 cm), (173.6 cm, 173.5 cm), (327.5 cm, 327.8 cm), (303.2 cm, 302.8 cm) and (353.6 cm, 353.4 cm), where *a* in (*a*, *b*) is the actual distance and *b* is the measured one. The maximum measurement error is 4 mm, indicating a very accurate distance measurement is achieved. Then, detection of two targets are demonstrated. The system diagram including the antenna pair and the targets is shown in Fig. 5(a), where the two metallic targets have the same size of 6 cm × 4 cm. Figure 5(b) shows the power spectrum of the de-chirped signal when the two targets are 87.4 cm and 188.0 cm away from the antenna pair, respectively. In Fig. 5(b), two obvious spectral peaks corresponding to the two targets are observed. The calculated distance corresponding to the two spectral peaks are 87.3 cm and 188.4 cm, with a measurement error of 1 mm and 4 mm, respectively. When the two targets are placed close to each other, the two spectral peaks have a small frequency spacing. Figure 5(c) shows the power spectrum of the de-chirped signal when the two targets are 3.8 cm away from each other. The frequency spacing between the two spectral peaks are 200 kHz, and the calculated distance between the two targets is 3.75 cm, which is very close to the actual value. According to (3), the range resolution *L*_RES_, or the minimum distance that can be distinguished between the two targets is 1.875 cm, corresponding to the situation that the spacing between the two spectral peaks equals to the repetition rate of the LFM signal. Figure 5(d) shows the power spectrum of the de-chirped signal when the two targets are placed 1.9 cm away from each other. Two spectral peaks with a spacing of 100 kHz are observed, indicating the two targets can be easily distinguished.

In the target detection experiment, the distance measurement error is related to the radar range resolution. Specifically, the spectrum calculated by performing FFT consists of a serial of discrete spectral lines with a frequency spacing that is equal to the repetition rate of the transmitted LFM signal. When the distance of a target changes within ± *L*_RES_/2, the spectral line with the maximum amplitude may not change position and the calculated distance remains unchanged, resulting in a measurement error no more than *L*_RES_/2. Therefore, when the echo signal reflected from the target is not covered by noise or other spectral spurs, the distance measurement error for the established 8-GHz radar is kept within 9.375 mm.

## Discussion

In the transmitter, the signal generation by photonic frequency multiplication has the potential to generate broadband LMF signals at a high central frequency. Although frequency quadrupling is adopted in the proposed radar, photonic frequency multiplication schemes with a multiplication factor as high as eighteen^18^ can be applied to further increase the bandwidth and central frequency of the generated LFM signals. Similar with electrical frequency multipliers, a photonic frequency multiplier also causes signal degradations. Firstly, the signal-to-noise ratio (SNR) may be degraded. In the experiment for target detection, in-band SNR of the electrical IF-LFM signal is measured to be 86 dB. After photonic frequency quadrupling, the in-band SNR measured after PD1 without electrical amplification is degraded to 72 dB. Secondly, the spurious property is degraded after frequency quadrupling. In the established system, the in-band spurious-free dynamic range (SFDR), which is the SNR when the in-band distortion equals to the noise floor, is measured to be 55 dBc and 49 dBc for the signal before and after frequency quadrupling, respectively. The signal degradations are due to the inherent signal deterioration in frequency multiplication as well as the signal deterioration in photonics-related operations such as optical-to-electrical conversion at a photodetector. In practice, these signal degradations would affect the radar detection range and accuracy. Another possible problem in the transmitter is the bias drift of the DPMZM, which can deteriorate the system stability. To achieve a long-term stabilized radar operation, bias control circuits should be applied^19^.

In the receiver, the RF conversion loss in de-chirp processing based on photonic frequency mixing is an important issue affecting the radar performance. In the established system, RF conversion loss of the photonic de-chirping module incorporating the optical amplifier is measured to be around 5 dB, which is comparable with the current electrical frequency mixers. The SFDR, measured by feeding the generated LFM signal to the photonic de-chirping module through an electrical cable, is about 48 dBc, which is close to that of the generated LFM signal in the transmitter. Therefore, the photonic de-chirping would not cause much performance deterioration. Another issue with the radar receiver is the use of OBPF. Theoretically, the OBPF is not required because the de-chirped signal obtained by beating the optical carriers at *f*_c_ + 2*f*_0_ + 2 *kt* and *f*_c_ + 2*f*_0_ + 2 *kt* + 4*k*Δ*τ* has the same frequency with that achieved by beating the carriers at *f*_c_ − 2*f*_0_ − 2*kt* and *f*_c_ − 2*f*_0_ − 2*kt* − 4*k*Δ*τ*. However, we suggest using the OBPF to improve the SNR of the de-chirped signal by suppressing the out-of-band amplitude noise, especially when an EDFA is applied after the PM. Here, the OBPF should have a sharp roll-off and a flat top to select out the desired optical frequencies if the minimum frequency of the LFM signal is small. In the experiment, the tunable OBPF has a minimum bandwidth of 50 pm (~6.25 GHz) and the side slop of the transmission edge is as high as 500 dB/nm, which is feasible for de-chirping of LFM signals with a minimum frequency no less than 5 GHz^20^.

In modern digital radar receivers, real-time signal processing at 500 MSa/s sampling rate is not a problem, thus real-time target detection can be realized by the proposed radar. When detecting a long range target, to ensure the de-chirped signal has a frequency within the real-time processing bandwidth of the receiver, chirp rate of the transmitted LFM signal can be reduced by adjusting the bandwidth and repetition rate of input IF-LFMCW signal, as demonstrated in Fig. 3. However, this may result in bandwidth reduction and thus degrade the range resolution. To address this problem, a photonic delay line, such as a span of optical fiber, can be inserted before the PM to introduce a known time delay to the reference optical signal. This time delay (denoted as Δ*τ’*) is used to cancel out part of the time delay (Δ*τ*) corresponding to the wireless transmission of the LFM signal, and hence a low-frequency de-chirped signal is achieved with Δ*f* = 4*k*(Δ*τ* − Δ*τ’*). When calculating the actual target distance, an extra distance of *c*Δ*τ’*/2 should be added. With this method, real-time processing is still realizable for long range target detection, as long as the transmitted signal power is large enough. Besides, by applying this photonic delay line technique to reduce the de-chirped frequency, the requirement for real-time target detection can be relaxed.

The range resolution of a radar is related to the operation bandwidth. Conventional K-band radars usually have a bandwidth of several hundreds of megahertz, and the range resolution is ~15 cm^21,22^. A terahertz radar can achieve a range resolution of ~1.5 cm^23^, but the performance is limited by the complex electric circuits and the short detection range. Photonics-based radar has the potential for a very broad operation bandwidth, which, however, has not been fully developed in previous demonstrations. For example, the photonics-based radar in^11^ has a maximum bandwidth of 200 MHz, corresponding to a range resolution of 7.5 m. In our experimental demonstration, the 8-GHz bandwidth is restricted by the bandwidth of the antenna pair. As for the proposed photonic signal generation and de-chirp processing, the operation bandwidth is only limited by the electro-optical modulators and photodetectors. Thus the proposed radar has the potential to be operated with a bandwidth of tens or even hundreds of gigahertz, making it possible to achieve an ultra-high-range-resolution below 1 cm.

## Conclusion

We have proposed and demonstrated a photonics-based real-time high-resolution radar applying optical signal generation and de-chirp processing within a compact configuration. The LFM signal generated by optical frequency quadrupling has a very large bandwidth that is required in a high-resolution radar. Besides, photonic de-chirping of the reflected echoes avoids the use of electrical frequency conversion and high-speed ADCs, making it possible for real time processing of a broadband signal in radar receivers. Performance of the proposed method is investigated through an established radar operating at K-band with an 8-GHz bandwidth. The experimental results confirm the feasibility and good performance of the proposed radar scheme, which is a promising solution for real-time ultra-high-range-resolution target detection.

## Methods

### Realization of optical Frequency-quadrupling

We assume the optical field fed into the DPMZM is *E*_in_(*t*) = *E*_c_cos(2*πf*_c_ *t*), where *E*_c_ is the amplitude of the optical field and *f*_c_ is the frequency of the optical carrier. The driving signal applied to the two sub-MZMs (MZM-a and MZM-b) is *V*_1_(*t*) = *V*_IF_cos(2*πf*_IF_ *t*) and *V*_2_(*t*) = *V*_IF_cos(2*πf*_IF_ *t* + *π/*2), respectively. Both MZM-a and MZM-b are biased at the maximum transmission point, and MZM-c is biased at the minimum transmission point. Under this condition, the optical field at the output of the DPMZM is^24^

4 $${E}_{{\rm{DPMZM}}}(t)=\frac{1}{2}{E}_{{\rm{c}}}\{\cos (2\pi {f}_{{\rm{c}}}t)\cos [m\,\cos (2\pi {f}_{{\rm{IF}}}t)]-\,\cos (2\pi {f}_{{\rm{c}}}t)\cos [m\,\cos (2\pi {f}_{{\rm{IF}}}t+\frac{\pi }{2})]\}$$

where the modulation index *m* equals to *πV*_IF_/2*V*_*π*_ with *V*_*π*_ being the half-wave voltage of the two sub-MZMs. Based on Jacobi-Anger expansions, (4) can be expanded to be

5 $${E}_{{\rm{DPMZM}}}(t)=-{E}_{{\rm{c}}}\sum _{n=1}^{\infty }{J}_{4n-2}(m)\times \{\cos [2\pi ({f}_{{\rm{c}}}+(4n-2){f}_{{\rm{IF}}})t]+\,\cos [2\pi ({f}_{{\rm{c}}}-(4n-2){f}_{{\rm{IF}}})t]\}$$

where *J*_n_ is the Bessel function of the first kind of order *n*. When *m* is within the typical range of (0, *π*), the optical sidebands with the order higher than 2 can be ignored without significant errors, so the optical field can be further simplified to be

6 $${E}_{{\rm{DPMZM}}}(t)=-{E}_{c}\{{J}_{2}(m)\cos [2\pi ({f}_{c}+2{f}_{{\rm{IF}}})t]+{J}_{2}(m)\cos [2\pi ({f}_{c}-2{f}_{{\rm{IF}}})t]\}$$

In equation (6), only the ± 2^nd^ order modulation sidebands exist. When this optical signal is sent to a photodetector, the obtained electrical signal has a frequency of 4*f*_IF_(*t*), and a frequency-quadrupled LFM signal is generated.

### Experimental components

The light source is a laser diode (TeraXion. Inc.) having a wavelength of 1550.51 nm and an output power of 16 dBm. The DPMZM (Fujitsu FTM7962EP) has a 3-dB bandwidth of 22 GHz and a half-wave-voltage of 3.5 V at 22 GHz. The phase modulator (EOSPACE Inc.) has a bandwidth of 40 GHz and a half-wave-voltage of about 3.5 V. An arbitrary waveform generator (Keysight 8195 A) with a maximum sampling rate of 65 GSa/s is applied to generate the IF-LFM signal. The electrical amplifiers (SHF 806E) used for boosting the power of the transmitted and received LFM signals have a 40-GHz bandwidth and a gain of 26 dB. The photodetector used for LFM signal generation (PD1) has a bandwidth of 40 GHz and the photodetector used for de-chirping (PD2) has a bandwidth of 10 GHz. The EDFA (Amonics Ltd. Mini EDFA) after the PM provides an optical power gain of about 10 dB. The OBPF is a tunable C-band optical filter (Yenista XTM-50) of which the bandwidth can be tuned form 50 pm to 800 pm and the side slop of the transmission edge is 500 dB/nm. The optical spectrum is measured by an optical spectrum analyzer (Yokogawa AQ6370C) with a resolution of 0.02 nm. A real-time oscilloscope (Keysight DSO-X 92504 A) is used to measure the waveform of the generated LFM signal with a sampling rate of 80-GSa/s. This oscilloscope is also used as the radar receiver for digitizing the de-chirped signal at a sampling rate of 500 MSa/s and performing real-time spectral analysis.
